# Post exposure prophylaxis coverage, vertical transmission and associated factors among hepatitis B exposed newborns delivered at Arsi zone health institutions, 2019

**DOI:** 10.1371/journal.pone.0238987

**Published:** 2020-10-14

**Authors:** Hinsermu Bayu, Bedasa Elias, Silashi Abdisa, Abdurhaman Tune, Husen Namo

**Affiliations:** 1 Department of Midwifery, College of Health Sciences, Arsi University, Mekelle, Ethiopia; 2 Department of Obstetrics and Gynaecology, College of Health Sciences, Arsi University, Mekelle, Ethiopia; 3 Department of Biomedical, College of Health Sciences, Arsi University, Mekelle, Ethiopia; 4 Department of Anaesthesia, College of Health Sciences, Arsi University, Mekelle, Ethiopia; Kaohsiung Medical University, TAIWAN

## Abstract

**Introduction:**

One third of the world population has been exposed to hepatitis B virus and an estimated 257 million people are chronically infected. The main route of hepatitis B virus (HBV) infection is vertical transmission. Post exposure prophylaxis is recommended by world health organization to have free Hepatitis B infection by 2030.

**Objective:**

The main purpose of this research project was to assess Hepatitis B virus post exposure prophylaxis coverage, rate of vertical transmission and factors among exposed newborns delivered at Arsi zone health institution.

**Methods:**

A cross-sectional study was conducted in Arsi zone health institutions among hepatitis B virus exposed newborns delivered at Arsi zone health institutions from January 2018 to September 2019. Systematic sampling technique was used to select 422 exposed newborns into the study. A pre-tested structured questionnaire and checklist were used to collect relevant data. Data was entered and cleaned using epidata7 & analyzed using SPSS version 25 software package. Both bivariate and multivariate analyses was carried out to identify associations. Odds ratio with 95% CI and P-value <0.05 was considered statistically significant.

**Results:**

The study revealed that among 401 exposed newborns only 83(20.7%), have been administered post exposure prophylaxis. But vertical transmission of hepatitis B virus (HBV) was observed in 32.4% (27.9%-36.9%) exposed newborns. Antenatal (ANC) attendance (AOR = .40, 95%CI = .23-.69), Instrumental delivery (AOR = 4.18, 95%CI = 2.05–8.51) HIV coinfection (AOR = 9.7, 95%CI = 4.37–21.34), Post exposure Prophylaxis (AOR = .20, 95%CI = .08-.50) and Knowledge on HBV (AOR = .27, 95%CI = .14-.53) are significant predictors of HBV vertical transmission.

**Conclusion:**

Magnitude of HBV post exposure prophylaxis coverage is very low while Rate of vertical transmission is high. Antenatal attendance, Instrumental delivery, Post exposure Prophylaxis and Knowledge on hepatitis B virus transmission are significant predictors of HBV vertical transmission.

## Introduction

Globally, one third of the population has been exposed to hepatitis B virus (HBV) and an estimated 257 million people are chronically infected. Approximately, there were 887,000 deaths, mostly from cirrhosis and hepatocellular carcinoma [1].

The main route of hepatitis B virus (HBV) infection is vertical transmission from hepatitis B virus infected mother to their exposed newborns. This vertical transmission occurs usually during birth or soon after birth following close contact [2]. Neonates who contract hepatitis will have an almost 90% risk of developing chronic HBsAg carriage and chronic liver disease. Infants may also spread the disease to siblings and to a community [3].

According to previous studies, vertical transmission rate ranges from 1% to 28% in developed countries where preventive measures are perfect [4]. But in sub-Saharan countries, hepatitis B virus (HBV) exposed newborns have up to 90% chance to become infected with the virus [5].

Maternal, social, demographic and obstetrical procedures like mode of delivery are known as important predictors of vertical transmission [6].

Post exposure prophylaxis offers best protective benefits to eradicate hepatitis B virus infection in new generation. World health organization recommended administration of three doses of HBV vaccine and two doses of immunoglobulin. The first doses of both vaccine and immunoglobulin has to be administered within 24 hours of birth. HBV vaccine has been reported to reduce the rate of vertical transmission from 90% to 5–10% [7].

Despite the presence of preventive measures, including availability of immune prophylaxis to exposed newborns and hepatitis B vaccine in Ethiopia, there are remaining threats to the successful eradication of HBV [8]. Therefore, the main purpose of this research project was to determine Coverage of post exposure prophylaxis, vertical transmission rate and its predictors among exposed newborns delivered at Arsi zone health institution.

## Methods and materials

A health facility based cross-sectional study was conducted in Arsi zone. from January 2018 to September 2019. Arsi zone is zone located at 165 km from Addis Ababa, the capital city of Ethiopia. It is divided into more than 130 kebeles (the smallest administrative unit) with a population estimated to be 2,637,657, of whom 1,323,424 are men and 1,314,233 women (Census 2007) [9].

The study population was comprised of all hepatitis B virus exposed newborns (newborns delivered to mothers who were already diagnosed HBV-positive) delivered at Arsi zone health institutions during the study period (January 2018 to September 2019). Participants were selected using systematic sampling technique based on chronological order of mothers admission to labor ward. The sample size was determined by using a single population proportion formula considering the following assumptions: post exposure prophylaxis coverage 50%, (**p** = 0.5), 5% level of significance (α = 0.05). The final sample size was adjusted for none response rate of 10% and it was 422.

Data was collected through face to face interview using a structured and pre-tested questionnaire developed from different literature. Data have been collected in two phases: **Phase one and phase two.** Phase one data collection was conducted on date of delivery. Interview of mothers and record review were made to assess mothers’ socio-demographic profile, some obstetrical factors. A 2ml of blood was drown from umbilical cord immediately After birth to assess newborn HBsAg status. **Phase two** data collection was held at 9^th^ month of delivery. Additional blood sample was collected from the exposed newborns Venus at 9^th^ month of birth during phase two data collection to determine hepatitis B surface antigen (HBsAg) serostatus.

Ten midwives and 10 lab technicians collected the data from January 2018 to September 2019. The data collection process was supervised by two MSc. midwives from Asella teaching hospital.

Two milliliters of umbilical cord and venous blood were collected at birth and 9^th^ month from the exposed new-borns by an experienced laboratory technologist, respectively. Afterwards, the blood was allowed to clot at room temperature for about 30 minutes as recommended by testing kit’s manufacturer (Dialab GmbH, Austria). The tube was centrifuged at 800g for 20 minutes; serum was separated; transferred into cryo-tube and finally stored in a freezer at -20˚C. Serological screening was done in the same day of collection and onsite using the Dialab1 HBsAg ELISA kit (Dialab GmbH, Austria). To maintain the quality of laboratory results, standard operating procedures (SOPs) of the test kit’s manufacturer was followed strictly. Positive and negative controls were run along with each batch of ELISA test kit [10].

Data was entered using epi7 and analyzed by SPSS version 25 software package. A p-value of ≤ 0.05 were used as a cut point to determine association in both bivariate and multi variate analysis. Tables, figures and text using frequency and summary statistics such as mean, standard deviation and percentage were used to present results. The degree of association between the independent and dependent variables was analyzed using odds ratio with 95% confidence interval.

Ethical clearance was obtained from Institutional Review Board (IRB) of Arsi University. A letter of cooperation was written to respective hospitals and health centers in the zone. Finally informed written consent was obtained from all mothers of the exposed newborns after they had been informed that their involvement is up to their willing. And they have been informed that they can withdraw any time. Mothers of the exposed newborns were also informed that data would be kept confidential using codes instead of any personal identifiers and is meant only for the purpose of the study. They have been also informed that they would not have any direct benefit because of their participation.

## Results

### Exposed new-borns’ mothers socio-demographic characteristics

Among the 422 sampled exposed new-borns majority of their mothers (401) responded to the questionnaire, giving a response rate of 95%. The mean age of their mothers was 28.6 years (28.6±5.8SD). The majority of their mothers 274 (68.3%) were Oromo, married (377, 94%) and orthodox Christian (188, 46.9%) (Table 1).

**Table 1:** Socio-demographic characteristics of mothers who gave birth to exposed new-borns at Arsi zone health institution, January 2018-September 2019.

Variables	Frequency(percentage)
**Age (**mean = 28.6, std = 5.8)	
13–22	66(16.5)
23–28	148(36.9)
29–34	111(27.7)
35–50	76(19.0)
**Marital status**	
Divorced	8(2.0)
Married	377(94.0)
Single	12(3.0)
Widowed	4(1.0)
**Religion**	
Muslim	162(40.4)
Orthodox	188(46.9)
Protestant	40(10.0)
Others*	11(2.7)
**Ethnicity**	
Amhara	79(19.7)
Gurage	36(9.0)
Oromo	274(68.3)
Others**	12(3.0)
**Mothers occupation**	
Housewife	178(44.4)
Government employee	87(21.6)
Self-employee	125(31.2)
Others***	11(2.7)
**Mothers education**	
College and above	70(17.5)
High school	25(6.2)
No formal education	52(13.0)
Primary education	166(41.4)
Secondary education	88(21.9)

Others

*: Wakefata,7^th^ day Adventist and Joba, Others

**: Tigre, Somali and zey, Others

***:- Student and daily labourer.

### Exposed new-borns mothers’ obstetrics and gynaecologic characteristics

Majority of the exposed newborns,257 (64.1%) delivered to mothers who gave birth at least once and 252 (62.8%) had ANC attendance for recent pregnancy. Two hundred sixty-six (66.3%) exposed newborns were delivered spontaneously while cesarean section accounts for 17.2%. On the other hand, 55 (13.7%) exposed newborns were delivered to mothers who were HBV and HIV co infected (Table 2).

**Table 2:** Exposed new-borns mothers obstetrics characteristics at Arsi zone health institution, January 2018-September 2019.

Variables	Frequency(percentage)
**Gravidity**	
I	120(29.9)
II- IV	206(51.4)
≥V	75(18.7)
**Parity**	
I	144(35.9)
II- IV	199(49.6)
≥V	58(14.5)
**Antenatal care follow up**	
No	149(37.2)
Yes	252(62.8)
**Mode of delivery**	
Cesarean section	69(17.2)
Instrumental delivery	66(16.5)
Spontaneous Vaginal Delivery	266(66.3)
**Episiotomy**	
No	261(65.1)
Yes	140(34.9)
**Antepartum Complication**	
No	299(74.6)
Yes	102(25.4)
**Maternal HIV status**	
Non-reactive	346(86.3)
Reactive	55(13.7)

### Knowledge on mode of transmission

**Fig 1:**
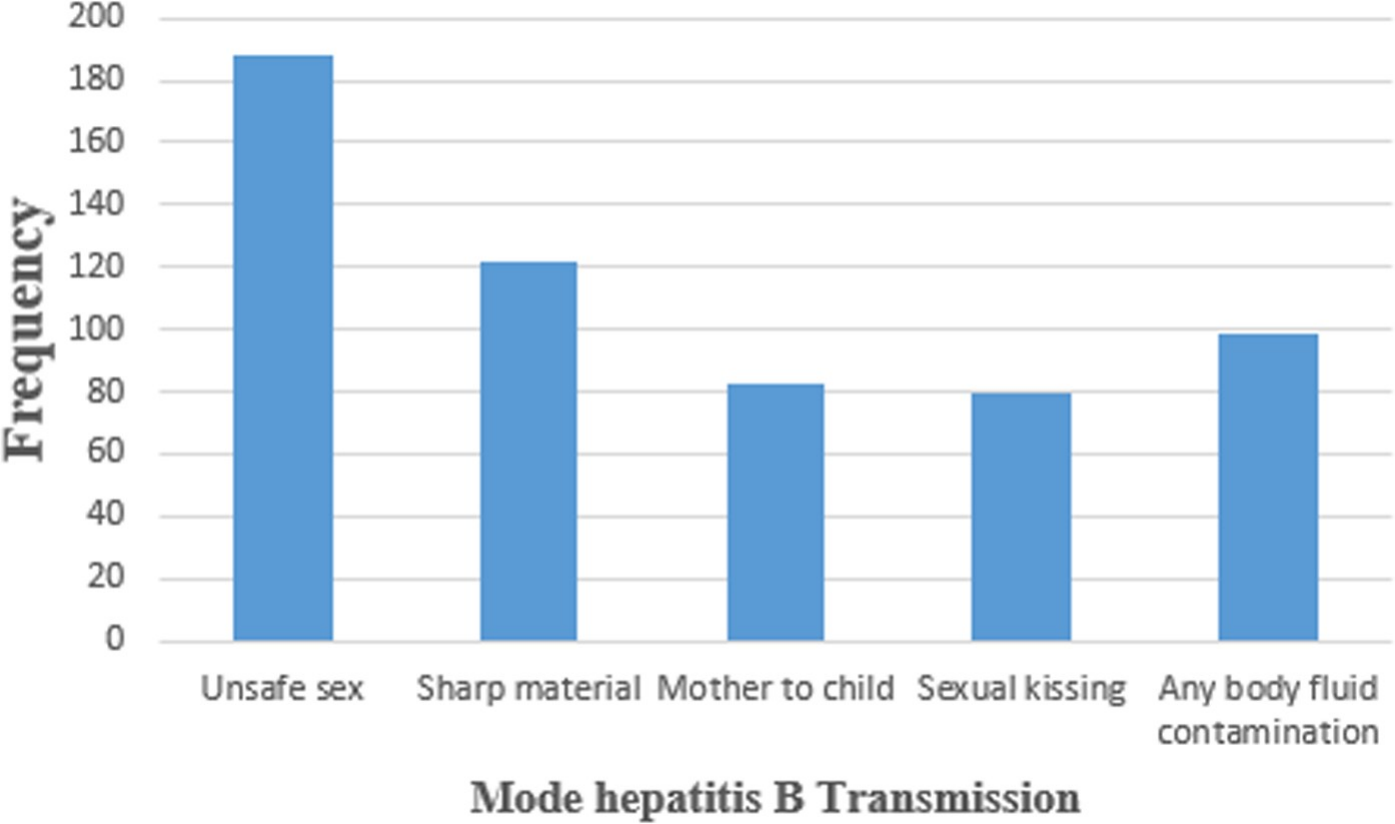
Mode of HBV infection mentioned by HBV positive mothers who gave birth to exposed new-borns at Arsi zone health institution, January 2018-September 2019. Only 165(41.1%) mothers are knowledgeable about hepatitis B virus infection in general and only 63(15.7%) took modern medicine for the viral (HBV) infection. Unsafe sexual transmission is the most commonly reported 188(32.9%) mode of transmission while mother to newborn transmission is reported only by 83(14.5%) mothers.

Only 165 (41.1%) mothers are knowledgeable about hepatitis B virus infection in general and only 63 (15.7%) took modern medicine for the viral (HBV) infection. Unsafe sexual transmission is the most commonly reported 188 (32.9%) mode of transmission while mother to newborn transmission is reported only by 83 (14.5%) mothers (Fig 1).

### Exposed new-borns characteristics

Among total exposed new-borns, 30 (7.5%) are preterm and 54 (13.5%) born with low birth weight. Four newborns (1.0%) born with first minute APGAR of less than four while 24 (6.0%) exposed newborns developed immediate complication such as fetal asphyxia (Table 3).

**Table 3:** Characteristics of exposed new-borns who born at Arsi zone health institution, January 2018-September 2019.

Variables	Frequency (percentage)
Gestational Age	
Preterm	30(7.5)
Term	293(73.1)
Post term	78(19.5)
**Birth weight**	
Low birth weight	54(13.5)
Normal birth weight	327(81.5)
Macrosomia	20(5.0)
**Time of Delivery**	
Day time	193(48.1)
Night time	208(51.9)
**First Minute APGAR**	
Low APGAR	4(1.0)
Intermediate	61(15.2)
Normal	336(83.8)
**Complication**	
No	377(94.0)
Yes	24(6.0)

### Post exposure prophylaxis coverage

Of the 401 exposed newborns, only 152 (37.9%) have been vaccinated. Furthermore, only 83 (54.6%) of the already vaccinated group have received Anti-hepatitis B immunoglobulin (HBIg). That means out of 401 exposed newborns, only 83 (20.7%) have received both vaccination and Anti-Hepatitis B immunoglobulin (Table 4).

**Table 4:** Post exposure prophylaxis coverage among exposed new-borns delivered at Arsi zone health institution, January 2018-September 2019.

Variables	Frequency (Percentage)
**Vaccination**	
No	249(62.1)
Yes	152(37.9)
**Timing of vaccination (n = 152)**	
Within 12 hrs	39(25.6)
Within 24 hrs	80(52.6)
Within 48 hrs	9(5.)
Within 72 hrs	24(15.9)
**Administration of HBIg (n = 152)**	
No	69(45.3)
Yes	83(54.7)
**Timing of HBIg administration (n = 83)**	
Within 12 hrs	40(48.2)
Within 24 hrs	29(35.0)
Within 48 hrs	4(4.8)
Within 72 hrs	10(12.0)

HBIg: Hepatitis B immunoglobulin.

### Vertical transmission rate

The overall vertical transmission rate was found to be 32.4% (27.9%-36.9%). In our follow up of mother-exposed newborn pairs, 22 (5.5%) infants became surface antigen positive since at birth. The rest, 108 (26.7%) became hepatitis B surface antigen positive between 9^th^ and 12th months.

### Factors associated with vertical transmission of hepatitis B virus

Bivariate analysis was done to assess any association between individual independent variables and vertical transmission of hepatitis B surface antigen. After controlling the effect of other variables; Antenatal care follow up, Mode of delivery, Post exposure prophylaxis, HIV coinfection and mother’s knowledge on mode of HBV transmission continued to be significantly associated with vertical transmission of HBsAg **(**P-values<0.05).

Exposed babies born form mothers who have ANC are about 60% less likely to be positive to HBsAg when compared to exposed babies born from non-ANC attendant (AOR = 0.397,95%CI = .228-.691). Exposed babies who underwent instrumental delivery are 4 times more likely to be positive HBsAg when compared with those who have been delivered spontaneously (AOR = 4.175, 95%CI = 2.046–8.517).

Babies who exposed to both Hepatitis B virus and HIV are about 10 times more like to be infected with HBsAg when compared with those exposed only to HBsAg(AOR = 9.665,95%CI = 4.374–21.335). On the other hand, Exposed babies who received post exposure prophylaxis are about 80% less likely to be infected with HBV when compared with those who did not received the post exposure prophylaxis (AOR = .202,95%CI = .081-.502).

Exposed babies born from mothers who are knowledgeable on HBV are about 72% less likely to be positive to HBsAg when compared to those of exposed babies born from non-knowledgeable mothers (AOR = .273, 95%CI = .141-.531) (Table 5).

**Table 5:** Bivariate and multivariate analysis of factors associated with hepatitis B virus vertical transmission among exposed newborns delivered at Arsi zone health institutions from January 2018 to September 2019.

Variables	HBsAg		Crude OR (95%CI)	Adjusted ORs(95%CI)
	Negative	Positive		
**Antenatal care follow up**				
No	83	66	1	1
Yes	188	64	.43(.28-.66)	**.40(.23-.69)**
**Gestational Age**				
Preterm	**26**	**4**	**1**	
Term	**202**	**91**	2.93(.99–8.63)	
Post term	**43**	**35**	5.29(1.69–16.60)	
**Birth Weight**				
Low birth weight	**47**	**7**	1	
Normal birth weight	**216**	**111**	3.45(1.51–7.88)	
Big baby	**8**	**12**	10.07(3.05–33.31)	
**Mode of Delivery**				
Cesarean section	**41**	**28**	2.11(1.21–3.68)	**1.55(.23–3.20)**
Instrumental	**29**	**37**	3.95(2.25–6.91)	**4.18(2.05–8.51)**
Spontaneous vaginal delivery	**201**	**65**	1	**1**
**Female genital Mutilation**				
No	**132**	**42**	1	
Yes	**139**	**88**	1.99(1.28–3.08)	
**Mother took Treatment for HBV**				
No	**217**	**121**	1	
Yes	**54**	**9**	.30(.14-.63)	
**HIV coinfection**				
No	**251**	**95**	1	**1**
Yes	**20**	**35**	4.62(2.54–8.41)	**9.7(4.37–21.34)**
**Newborn Vaccination**				
No	**154**	**95**	1	
Yes	**117**	**35**	.49(.31-.77)	
**Newborn HBIg administration**				
No	**196**	**122**	1	**1**
Yes	**75**	**8**	.17(.08-.37)	**.20(.08-.50)**
**Mother’s Knowledge**				
Not Knowledgeable	**132**	**104**	1	**1**
Knowledgeable	**139**	**26**	.24(.15-.39)	**.27(.14-.53)**

## Discussion

The study has shown that only 83 (20.7%) of exposed newborns have received post exposure prophylaxis. The current post exposure prophylaxis coverage (20.7%) is much lower than results from large scale study done in Greenland, where 80% of exposed newborns received at least birth dose vaccination [11]. Another study with the same design has showed 99.4% of babies born to HBsAg positive mothers has received birth dose vaccination [12]. HBIG coverage in western areas of Shandong Province is 81.1% among infants with known HBsAg-positive mothers [13].

This wide gap in vaccination coverage might be due to socio-economic differences among study groups. Poor maternal knowledge on HBV could also be contributing for poor birth dose vaccination and prophylaxis coverage. Although the WHO recommends commencing HBV vaccination at birth, in Ethiopia there is high vaccine availability interruption and immunoglobulin in accessibility and costly.

Another important result from this study is rate of hepatitis B virus vertical transmission. Thirty-two-point four percent (32.4%) of exposed newborns become positive to HBsAg at 12^th^ month of delivery irrespective of post exposure prophylaxis coverage.

The current result is equivalent with the same studies done in North America and South Africa where vertical transmission rate among exposed newborns is 34%,28% respectively [14]. But our finding is higher than most of results from different parts of developed countries, which ranges from 1.1%-18% [15–18]. Not only that, the current vertical transmission of HBV is much higher than results from studies done in eastern region (5.9%) and Kumasi provinces (8%) of Ghana [19, 20]. The high vertical transmission rate could be explained by very low post exposure prophylaxis coverage among newborns delivered to HBsAg positive mothers.

From this longitudinal follow up study it was found that Antenatal care is one of the significant predictors of HBV vertical transmission. Exposed newborns delivered to mothers with antenatal care is 60% less likely to be positive for HBsAg when compared to exposed newborns delivered to non ANC attendant mothers (AOR = **.40,95%CI = .23-.6**9). The finding is congruent with other results that ANC is an opportunity to promote healthy behaviors such as vaccination, immunoglobulin administration immunization and institutional child birth [21].

Mode of delivery is also associated with vertical transmission rate of HBV. Exposed newborns delivered by instrument (vacuum or forceps) are 4 times more likely to be positive to HBsAg when compared to those of exposed newborns delivered spontaneously (AOR = **4.18,95%CI = 2.05–8.51)**. But this result is inconsistent with comparative study, which reports, there is no difference on vertical transmission rate of HBV by mode of delivery [22, 23]. This inconsistencies whether Mode of delivery on HBV vertical transmission rate could be explained by other variables like type cesarean section, type of instrumental delivery and membrane status. It is obvious that ANC attendant will get information on route of HBV transmission and means to control it.

In this study, HIV coinfection is found seriously affecting hepatitis B virus vertical transmission rate. Babies born to mothers who are positive for both HBV and HIV is about 10 time more likely positive to HBsAg when compare to babies born to mothers positive to only HBV(AOR = 9.7,95%CI = 4.37–21.34). This result is consistent with the findings of studies done in Burkina Faso, maternal HIV–HBV co-infection increased the probability of MTCT of HBV by 2 [24]. Our result is confirmed by Sub-Saharan African studies where, vertical transmission occurs twice among HIV–HBV co-infected mothers [25]. Further more Exposed babies who received both Vaccination and immunoglobulin immediately after birth are 80% less likely to become positive for HBsAg compared to non-vaccinated and immunized one (AOR = .20, 95%CI = .08-.50). The same result was reported by previous findings from strong design, vaccine plus hepatitis B immunoglobulin reduced hepatitis B occurrence by 59% [26, 27].

The current research showed mother’s knowledge is also implicated in HBV vertical transmission. Exposed babies born from mothers’ who are knowledgeable about route of HBV transmission are 63% less likely to be infected with the HBV when compared to those exposed newborns delivered from non-knowledgeable mothers **(**AOR = .27, 95%CI = .14-.53) The above association could be explained by that knowledge is universally accepted factors in affecting preventive health care utilization. The more mothers are knowledgeable about route of transmission for HBV, the greater her newborn will be both vaccinated and immunized.

## Conclusion

Significant number of exposed new-borns are still acquiring hepatitis B virus from their mother due to poor post exposure prophylaxis coverage. Antenatal attendance, Instrumental delivery, Post exposure Prophylaxis and Knowledge on hepatitis B virus transmission are significant predictors of HBV vertical transmission.
